# Clinical Approach to Hypocalcemia in Newborn Period and Infancy: Who Should Be Treated?

**DOI:** 10.1155/2019/4318075

**Published:** 2019-06-19

**Authors:** Dogus Vuralli

**Affiliations:** Hacettepe University, Faculty of Medicine, Department of Pediatrics, Division of Pediatric Endocrinology, Ankara, Turkey

## Abstract

**Introduction:**

Hypocalcemia is a common metabolic problem in newborn period and infancy. There is consensus on the treatment of the symptomatic cases while the calcium level at which the treatment will be initiated and the treatment options are still controversial in asymptomatic hypocalcemia.

**Methods:**

This review article will cover hypocalcemia with specific reference to calcium homeostasis and definition, etiology, diagnosis, and treatment of hypocalcemia in newborn and infancy period.

**Results:**

Hypocalcemia is defined as total serum calcium <8 mg/dL (2 mmol/L) or ionized calcium <4.4 mg/dL (1.1 mmol/L) for term infants or preterm infants weighing >1500 g at birth and total serum calcium <7 mg/dL (1.75 mmol/L) or ionized calcium <4 mg/dL (1 mmol/L) for very low birth weight infants weighing <1500 g. Early-onset hypocalcemia is generally asymptomatic; therefore, screening for hypocalcemia at the 24th and 48th hour after birth is warranted for infants with high risk of developing hypocalcemia. Late-onset hypocalcemia, which is generally symptomatic, develops after the first 72 h and toward the end of the first week of life. Excessive phosphate intake, hypomagnesemia, hypoparathyroidism, and vitamin D deficiency are commonest causes of late-onset hypocalcemia. Hypocalcemia should be treated according to etiology. Calcium replacement is the cornerstone of the treatment. Elementary calcium replacement of 40 to 80 mg/kg/d is recommended for asymptomatic newborns. Elementary calcium of 10 to 20 mg/kg (1–2 mL/kg/dose 10% calcium gluconate) is given as a slow intravenous infusion in the acute treatment of hypocalcemia in patients with symptoms of tetany or hypocalcemic convulsion.

**Conclusion:**

Since most infants with hypocalcemia are usually asymptomatic, serum total or ionized calcium levels must be monitored in preterm infants with a gestational age <32 weeks, small for gestational age infants, infants of diabetic mothers, and infants with severe prenatal asphyxia with a 1 min Apgar score of <4. The treatment of hypocalcemia should be initiated immediately in infants with reduced calcium levels while investigating the etiology.

## 1. Introduction

Hypocalcemia is a common metabolic problem in newborn period and infancy. There is consensus on the treatment of the symptomatic cases while the calcium level at which the treatment will be initiated and the treatment options are still controversial in asymptomatic hypocalcemia. This review article will cover hypocalcemia with a specific emphasis on calcium homeostasis and definition, etiology, diagnosis, and treatment of hypocalcemia in newborn and infancy period.

## 2. Role of Calcium in Humans

Calcium is involved in many biochemical processes in the body, such as blood coagulation, intracellular signal transduction, neural transmission, muscle functions, cellular membrane integrity, and function, cellular enzymatic activities, cell differentiation, and bone mineralization [1, 2]. About 99% of body calcium resides in the bone tissue and the remaining is present in the extracellular fluid [3]. Almost half of the calcium found in the extracellular fluid is in the ionized active form, whereas 10% is complexed to anions such as phosphate, citrate, sulfate, and lactate, and 40% is bound to albumin [4, 5].

## 3. Factors Affecting Serum Calcium Level

Serum calcium level is affected from the serum levels of phosphate, magnesium, albumin, and bicarbonate. The change in albumin concentration does not change the blood level of ionized calcium but changes measurement of the total calcium level. In general, the plasma calcium concentration reduces by 0.8 mg/dL for every 1.0 g/dL reduction in plasma albumin level [6]. When ionized calcium, the active form, is not measured directly, calcium corrected for the measured total calcium and measured albumin should be calculated. Furthermore, the blood pH affects the level of ionized calcium. Alkalosis increases the amount of albumin-bound calcium and decreases the level of ionized calcium, leading to symptoms of hypocalcemia [7]. In acute respiratory alkalosis, the level of ionized calcium falls to 0.16 mg/dL for each 0.1 unit increase in pH [7]. Conversely, in metabolic acidosis, calcium-albumin binding is reduced and the level of ionized calcium increases. Magnesium is also involved in the regulation of intracellular release of parathyroid hormone. Low magnesium may reduce the activity of parathyroid hormone or cause resistance to parathyroid hormone (PTH), and long-term magnesium deficiency inhibits the release of PTH [8].

Calcium homeostasis is maintained by hormones, such as PTH, calcitonin, vitamin D, and calcium-sensing receptors. PTH increases bone resorption and, consequently, the serum level of calcium. In the kidneys, PTH increases the activity of 1-*α*-hydroxylase in the proximal tubules, increasing production of the active form 1,25-dihydroxyvitamin D from 25-hydroxyvitamin D. Furthermore, PTH increases phosphate excretion, calcium, and magnesium reabsorption in the distal tubules. The active form of vitamin D acts on the bones, intestines, and parathyroid glands. It increases osteoid mineralization in the bones and causes resorption at high doses. 1,25-Dihydroxyvitamin D also increases the intestinal absorption of calcium and phosphate ions and decreases the renal excretion of these ions. Vitamin D inhibits PTH secretion by the parathyroid glands. Calcitonin primarily reduces bone resorption, promotes calcium deposition in the bone by mineralization, and, consequently, decreases the serum calcium level. Calcitonin also increases the renal excretion of calcium and phosphate ions and decreases the gastrointestinal absorption of these ions [9].

## 4. Fetal and Neonatal Calcium Homeostasis

Calcium is transferred from the maternal circulation to the fetal circulation by active transport from the placenta in the last trimester. Thus, the calcium concentration is higher in cord blood than in maternal blood at delivery. At term pregnancy, fetal total and ionized calcium concentrations are 10–11 mg/dL (2.5–2.75 mmol/L) and 6 mg/dL (1.5 mmol/L), respectively, in umbilical cord blood [10]. PTH and calcitonin cannot pass through the placental barrier. Parathyroid hormone-related peptide is the major regulator of positive calcium balance in the placenta [11].

The postpartum serum level of calcium in newborns is associated with various factors such as PTH secretion, dietary calcium intake, renal calcium reabsorption, skeletal calcium stores, and vitamin D status. After the infant becomes detached from the placenta in the postpartum period, serum total and ionized calcium levels decrease, reaching a physiological nadir in a healthy 2-day term infant. By contrast, phosphate levels increase. The pace and amount of such decrease in calcium levels are inversely related to the gestational week [12]. Such a decreased level of calcium is associated with hypoparathyroidism, nonresponsiveness of target organs to PTH, vitamin D metabolism disorders, hyperphosphatemia, hypomagnesemia, and hypercalcitonemia in the first days of life [12]. PTH secretion increases in the first 48 h of life and with the increased PTH secretion, the intestinal absorption of calcium and phosphate, the renal reabsorption of calcium, and the renal excretion of phosphate increase in the newborn. Similarly, serum calcium levels start increasing and serum phosphate levels start decreasing. Within the first four weeks of birth, the intestinal absorption and renal reabsorption of calcium become mature [13].

## 5. Definition of Hypocalcemia 

Hypocalcemia is defined as total serum calcium <8 mg/dL (2 mmol/L) or ionized calcium <4.4 mg/dL (1.1 mmol/L) for term infants or preterm infants weighing >1500 g at birth and total serum calcium <7 mg/dL (1.75 mmol/L) or ionized calcium <4 mg/dL (1 mmol/L) for very low birth weight infants weighing <1500 g [14]. The main clinical symptoms of hypocalcemia include apnea, cyanosis, poor feeding, vomiting, tachycardia, heart failure, prolonged QT interval, irritability, tremor, laryngospasm, tetany, hyperacusia, jerking and twitching episodes, and focal and generalized seizures [15]. Early-onset neonatal hypocalcemia is mostly asymptomatic.

## 6. Etiology of Hypocalcemia in the Newborn Period and Infancy

Based on the time of onset the hypocalcemia defined as early-onset or late-onset hypocalcemia.

### 6.1. Early-Onset Hypocalcemia

It generally presents within the first 72 h of life. Early-onset hypocalcemia is caused by an increased reduction in the serum calcium level that physiologically occurs within the first three days in newborns, and delayed PTH secretion in response to hypocalcemia [13]. Early-onset neonatal hypocalcemia is more common in preterm infants, infants with intrauterine growth retardation, infants with perinatal asphyxia, and the infants of diabetic mothers.

Approximately one-third of preterm infants and most of the very low birth weight infants have low serum calcium levels during the first 48 h of life [12]. The causes of hypocalcemia in premature infants include early discontinuation of calcium transfer through the placenta, an exaggerated decrease in the serum calcium level that physiologically occurs postpartumly, the reduced response of target organs to PTH, and increased calcitonin levels [16]. The main causes of hypocalcemia in infants with asphyxia include increased phosphate load due to cellular damage, increased calcitonin production, renal failure, and decreased PTH secretion [16, 17]. The main cause of hypocalcemia in the infants of diabetic mothers is hypomagnesemia in the mother and the infant due to increased maternal urinary excretion of magnesium during pregnancy. Hypomagnesemia causes functional hypoparathyroidism in the infant [18]. The increased maternal calcium due to maternal hyperparathyroidism passes to the infant through the placenta and suppresses fetal PTH synthesis and impairs PTH response to postpartum hypocalcemia. PTH suppression, in this case, can be severe and lead to convulsions in the early neonatal period and may persist for months [19]. Mothers may be asymptomatic for hypercalcemia. Therefore, in course of early-onset neonatal hypocalcemia, the maternal serum levels of calcium and PTH must be measured for etiological evaluation.  Table 1 presents the causes of early-onset neonatal hypocalcemia.

### 6.2. Who Should Be Screened for Hypocalcemia?

Since most infants with early-onset hypocalcemia are usually asymptomatic, serum, preferably ionized, calcium should be measured in infants having risk factors for hypocalcemia. Preterm infants with a gestational age <32 weeks, infants of diabetic mothers, and infants with severe prenatal asphyxia and a 1 min Apgar score of <4 who are at risk for hypocalcemia should be screened at 24 and 48 h after birth [20]. For infants with an extremely low birth weight (birth weight <1000 g), calcium levels should be measured at 12, 24, and 48 h of birth. For preterm infants with a birth weight of 1000–1500 g, calcium level is measured at 24 and 48 h of birth. Monitoring of calcium levels should continue until values return to normal and calcium intake is adequate.

### 6.3. Late-Onset Hypocalcemia

Late-onset hypocalcemia, which is usually symptomatic, occurs after the first 72 h and generally by the end of the first week of birth. The most common causes of late-onset hypocalcemia include excessive phosphate intake, hypomagnesemia, hypoparathyroidism, and vitamin D deficiency [21–23]. Feeding with cow milk may cause hyperphosphatemia, leading to hypocalcemia [24]. Hypomagnesemia can cause impaired PTH secretion and reduced peripheral response to PTH, leading to hypocalcemia [25]. The causes of neonatal deficiency of vitamin D include maternal deficiency of vitamin D, malabsorption, renal failure, and hepatobiliary diseases of infants. In vitamin D deficiency, hypocalcemia is usually accompanied by hypophosphatemia. If hypocalcemia is accompanied by hyperphosphatemia, then hypoparathyroidism should be considered as the most common cause. Hypoparathyroidism can be primary or secondary. Primary hypoparathyroidism can be isolated or associated with syndromes such as DiGeorge syndrome [26]. Additionally, activating mutations of calcium-sensing receptors may cause primary hypoparathyroidism with autosomal dominant inheritance [27]. In such mutations, potassium channel functions in the kidneys are inhibited, leading to a Bartter-like syndrome, hypopotasemic metabolic alkalosis, hyperreninemia, hyperaldosteronism, and hypercalciuric hypocalcemia [28]. The most common conditions among syndromic primary hypoparathyroidism include the DiGeorge syndrome which is characterized with hypoplasia of the parathyroid glands, thymic hypoplasia, congenital heart diseases, and facial anomalies, and CATCH 22 syndrome with the cardiac anomaly, facial anomaly, asymmetric crying face, thymic hypoplasia, cleft palate, hypothyroidism, and deletion in chromosome 22 [29]. PTH resistance of pseudohypoparathyroidism may be transient as in the case of renal dysplasia or obstructive uropathy or permanent because of* GNAS* mutations. Pseudohypoparathyroidism type 1a (Albright's hereditary osteodystrophy) due to* GNAS* mutations involves generalized hormone resistance (PTH, TSH, FSH, and LH), short stature, and various skeletal anomalies [30]. However, skeletal symptoms that are typical for the later periods are not usually observed in the neonatal period. Pseudohypoparathyroidism type 1b does not involve skeletal symptoms but does resistance to PTH and TSH; and urinary cAMP increases after PTH administration, though phosphaturia does not occur [31]. The causes of late-onset hypocalcemia are summarized in Table 1.

## 7. Approach to Hypocalcemia in the Newborn Period and Infancy

A detailed history including pregnancy and family history should be obtained in cases of hypocalcemia in the newborn period and infancy. Pregnancy history should be questioned, especially in cases of gestational diabetes, toxemia of pregnancy, and maternal deficiency of vitamin D. Furthermore, questioning should include conditions that may be associated with early-onset hypocalcemia such as prematurity, low birth weight, asphyxia, neonatal sepsis, history of using medication for the mother and the infant, formula feeding status, phosphate load of the formula (if used), blood transfusion history, and presence of maternal hyperparathyroidism. The family history should be obtained for genetic diseases causing hypocalcemia in the newborn period and infancy. The physical examination should be made after considering the presence of any facial anomalies, cleft palate, and asymmetric crying face in terms of syndrome-related hypocalcemia. Among blood tests, ionized calcium, phosphate, alkaline phosphatase, magnesium, albumin, and creatinine levels in addition to serum total calcium level should be measured. Measured ionized calcium provides information on the active form of calcium. Albumin-calcium binding increases and the ionized calcium level decreases in metabolic or respiratory alkalosis. Low albumin suggests pseudohypocalcemia; and calcium measurement must be corrected for albumin levels. The levels of PTH, 25-hydroxyvitamin D, and, if necessary, 1,25-dihydroxyvitamin D, urine calcium, and creatinine should be measured. For newborns with hypercalciuria despite hypocalcemia, familial hypercalciuric hypocalcemia (activating mutation of calcium-sensing receptors) should be considered. In case of hypocalciuria, measurement of serum phosphate and PTH could be diagnostic. A low or inappropriately normal PTH despite hypocalcemia may suggest primary hypoparathyroidism or hypomagnesemia. Hypoparathyroidism can be transient, associated with maternal hyperparathyroidism, or permanent. In hypoparathyroidism, the serum calcium level decreases, whereas the serum phosphate level increases. If the PTH level is elevated in response to hypocalcemia, the parathyroid glands are considered working normally, and serum phosphate levels should be evaluated for differential diagnosis. If the serum phosphate level is low, then diseases of vitamin D metabolism should be considered. In such diseases, hypophosphatemia accompanies hypocalcemia and elevated alkaline phosphatase levels. The PTH level is elevated in response to hypocalcemia, and calcium and phosphate absorption is impaired. In such cases nutritional deficiency of vitamin D or malabsorption of vitamin D or a genetic disorder of vitamin D metabolism may be the underlying etiology. In cases with elevated serum phosphate levels and increased PTH levels in response to hypocalcemia, excessive exogenous intake of phosphate, renal failure, and pseudohypoparathyroidism (PTH resistance) should be considered. In such cases, serum creatinine and blood urine nitrogen levels should be measured for evaluation of renal failure. Typical skeletal symptoms are not present in cases with neonatal pseudohypoparathyroidism and* GNAS* analysis is required for diagnosis in such cases. Hypomagnesemia is another cause of hypocalcemia and can be due to renal magnesium loss due to osmotic diuresis, tubulopathy, or diuretic use, or gastrointestinal magnesium loss, even if it is rare (chronic diarrhea, malabsorption). The serum magnesium level should be measured especially in cases with no response to hypocalcemia treatment. Other causes of hypomagnesemia are maternal magnesium deficiency, maternal diabetes, intrauterine growth retardation, intestinal magnesium transport disorders, infantile isolate renal magnesium loss, nephrocalcinosis, and hypercalciuria [32, 33]. There is a state of functional hypoparathyroidism in hypomagnesemia.

**Figure 1:**
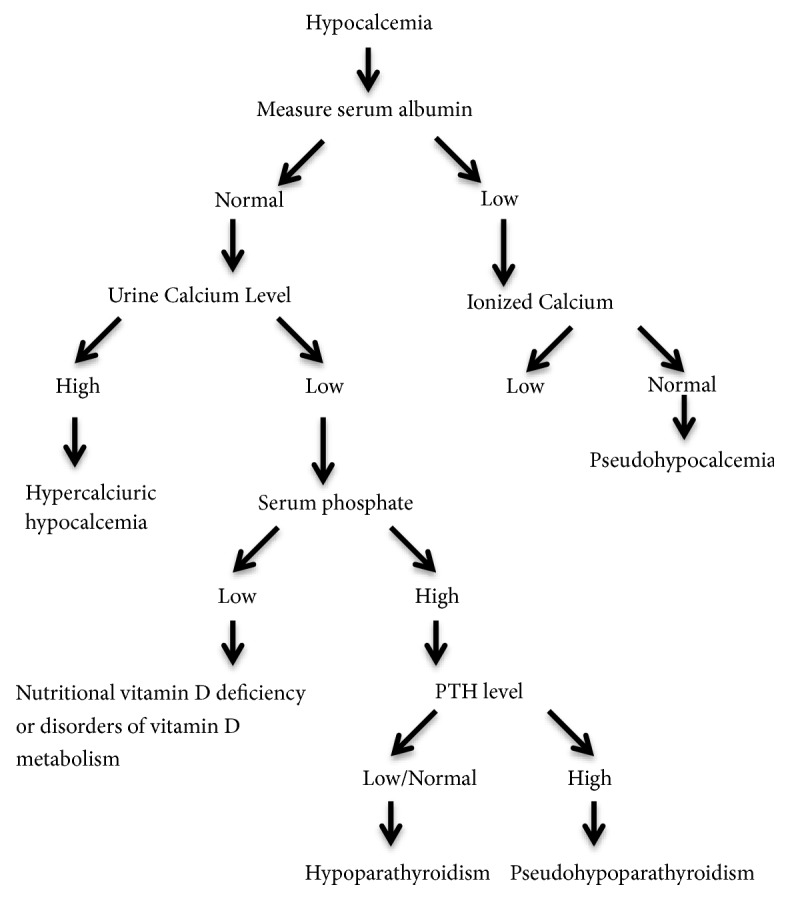
Diagnostic Approach to Hypocalcemia in Newborn Period and Infancy. *∗*Modified from Root AW, Diamond FB. (2008) Disorders of Mineral Homeostasis in the Newborn, Infant, Child, and Adolescent. In: Sperling MA, editor.* Pediatric Endocrinology*, 3rd ed. Philadelphia: Saunders/Elsevier. p. 686-769.

Electrocardiography (ECG) should be performed to establish the effect of severe hypocalcemia on tissues and the heart. In cases of hypocalcemia, ECG may reveal prolonged QT interval, QRS, and ST changes and ventricular arrhythmia [34]. Knee X-ray may help in diagnosing rickets in infancy period. Chest radiography can be used to evaluate the presence of a thymic shadow in cases with suspected DiGeorge syndrome. Furthermore, immunological methods can be used to determine the T-cell function in the DiGeorge syndrome. In such cases, a reduced count of CD4 lymphocytes is detected in addition to dysmorphic facial appearance, and fluorescence in situ hybridization (FISH) analysis can be used to show microdeletion in the chromosome 22q11.2 [35]. The diagnostic approach to hypocalcemia in the newborn period and infancy is summarized by an algorithm in Figure 1.

## 8. Treatment of Hypocalcemia in the Newborn Period and Infancy

The cornerstone of treatment of hypocalcemia is calcium replacement and the treatment options may vary by symptoms and the extent of hypocalcemia. Early-onset hypocalcemia is usually asymptomatic and treatment is recommended when the serum calcium level is <6 mg/dL in preterm and 7 mg/dL in term infants [35]. It is recommended administering 40 to 80 mg/kg/d elemental calcium replacement for asymptomatic newborns [20]. For infants who require parenteral nutrition, calcium can be added as 10% calcium gluconate (500 mg/kg/d, 50 mg/kg/d of elemental calcium) and given in continuous infusion. If parenteral calcium is administered for >2 days, phosphorus should also be replaced based on serum phosphate levels. In newborns with symptoms such as tetany or convulsion, intravenous 10 to 20 mg/kg of elemental calcium (1–2 mL/kg/dose 10% calcium gluconate) is administered by slow infusion for about 10 min under cardiac monitoring for the acute treatment of hypocalcemia. This treatment does not normalize the calcium level but it prevents the severe symptoms of hypocalcemia, such as convulsion. Following the administration of calcium as a bolus, 50 to 75 mg/kg/d or 1 to 3 mg/kg/h elemental calcium infusion should be initiated [13]. Continuous calcium gluconate infusion is preferred rather than 1 mL/kg/dose intravenous bolus doses every 6 h. The amount of calcium given should be adjusted by measuring calcium every 8 to 12 h until normal calcium values are achieved. Severe tissue necrosis may occur due to extravasation of calcium in the intravenous calcium gluconate therapy. Therefore, appropriate vascular access should be ensured and the infusion rate should not exceed 1 mg/min. Cardiac arrhythmias such as bradycardia may occur and even cardiac arrest may develop during calcium gluconate infusion; therefore, intravenous administration should be performed slowly for 10 to 30 min under cardiac monitoring. If an umbilical venous catheter is used for calcium administration, then the catheter tip should be in the inferior vena cava; a catheter tip in the portal vein may cause hepatic necrosis.

In patients who are asymptomatic or have mild symptoms or who have achieved normocalcemia by intravenous calcium, oral calcium therapy can be administered. In such patients, calcium lactate, carbonate, or citrate may be used and 40 to 80 mg/kg/d of elemental calcium can be administered in 3 to 4 doses. After the calcium level returns to normal, serum and urine levels of calcium and creatinine should be evaluated at frequent intervals and the dose should be adjusted so that daily urinary calcium excretion will be <4 mg/kg/d. Complications such as iatrogenic hypercalcemia, nephrocalcinosis, and renal failure can thus be avoided.

In patients with vitamin D deficiency, 1000 to 2000 IU/d of vitamin D replacement is recommended. Since the production of active vitamin D is impaired in vitamin D metabolism disorders or hypoparathyroidism, active vitamin D preparations (20–60 ng/kg/d calcitriol) should be administered. The treatment of hypoparathyroidism should aim to keep the serum calcium level at the lower limit of normal to avoid hypercalciuria and nephrocalcinosis. By contrast, the treatment goal in pseudohypoparathyroidism is to normalize PTH levels by keeping the serum calcium level close to the upper limit of normal.

No response can be achieved in hypocalcemia treatment without treating hypomagnesemia first. Magnesium sulfate (25–50 mg/kg or 0.2–0.4 mEq/L per dose every 12 h, intravenously over 2 h or intramuscularly) should be administered until serum magnesium concentration rises above 1.5 mg/dL (0.62 mmol/L). Rapid intravenous infusion of magnesium must be avoided because of the risk of arrhythmias. In infants with excessive phosphate load, the treatment goal is to reduce the serum phosphate level. Therefore, high-phosphate feeding should be avoided and infants should be fed with a diet high in calcium and low in phosphate either with human milk or a formula with low phosphate content. Calcium salts can also be helpful in decreasing the serum phosphate level.

## 9. Conclusion

Hypocalcemia is a common metabolic problem in newborns. Since most infants with hypocalcemia are usually asymptomatic, serum total or ionized calcium levels must be monitored in preterm infants with a gestational age <32 weeks, small for gestational age infants, infants of diabetic mothers, and infants with severe prenatal asphyxia. The treatment of hypocalcemia should be initiated immediately in infants with reduced calcium levels while investigating the etiology.

## Figures and Tables

**Table 1:** Causes of early and late onset hypocalcemia in the newborn period and infancy.

Early-onset hypocalcemia	Late-onset hypocalcemia
(i) Prematurity	(i) Increased phosphate load (feeding with cow milk, feeding with high-fiber formula, renal failure)
(ii) Intrauterine growth retardation (low birth weight)	(ii) Hypomagnesemia
(iii) Preeclampsia	(iii) Vitamin D deficiency
(iv) Asphyxia	(iv) PTH resistance (pseudohypoparathyroidism)
(v) Sepsis	(v) Hypoparathyroidism
(vi) Infants of diabetic mothers	(a) Primary hypoparathyroidism
(vii) Severe maternal deficiency of vitamin D	(1) Isolated hypoparathyroidism
(viii) Maternal hyperparathyroidism	(2) CaSR activating mutations (hypercalciuric hypocalcemia)
(ix) Mother using anticonvulsants (phenytoin sodium, phenobarbiturate)	(3) Syndromic hypoparathyroidisms (DiGeorge syndrome, CATCH-22, Kenny-Caffey syndrome, Barakat syndrome, Kearns-Sayre syndrome, Pearson syndrome)
(x) Maternal intake of high-dose antacids	(b) Secondary hypoparathyroidism (maternal hyperparathyroidism)
(xi) Use of aminoglycosides and anticonvulsants in the newborn	(vi) Iatrogenic
(xii) Iatrogenic (alkalosis, use of blood products, lipid infusions and diuretics, phototherapy)	(a) Use of citrate-blood products
	(b) Lipid infusions
	(c) Bicarbonate therapy
	(d) Loop diuretics (furosemide)
	(e) Phosphate therapy

*∗*Modified from Root AW, Diamond FB. (2008) Disorders of Mineral Homeostasis in the Newborn, Infant, Child, and Adolescent. In: Sperling MA, editor. *Pediatric Endocrinology*, 3rd ed. Philadelphia: Saunders/Elsevier. p. 686-769; from Carpenter TO. (2006) Neonatal hypocalcemia. In: Favus MJ, editor. *Primer on the Metabolic Disease and Disorders of Mineral Metabolism,* 6th ed. Washington, D.C. American Society for Bone and Mineral Research. p. 224-227.
